# Prevalence of malnutrition among settled pastoral Fulani children in Southwest Nigeria

**DOI:** 10.1186/1756-0500-1-7

**Published:** 2008-03-12

**Authors:** Uwem F Ekpo, Akin M Omotayo, Morenike A Dipeolu

**Affiliations:** 1 Department of Biological Sciences, University of Agriculture, Abeokuta, Nigeria; 2 Department of Agricultural Extension and Rural Development, University of Agriculture, Abeokuta, Nigeria; 3 Department of Veterinary Public Health, College of Veterinary Medicine, University of Agriculture, Abeokuta, Nigeria

## Abstract

**Background:**

There is a dearth of information on the health of pastoral Fulani children living in southwestern Nigeria. These are fully settled pastoralists whose economy are centred on cattle and farming. In other to monitor and plan appropriate nutritional intervention for their children, a cross-sectional study was carried out to determine the prevalence of malnutrition of pastoral Fulani children.

**Findings:**

Fulani's children aged 6 months to 15 years, living in 61 settlements in Kwara, Ogun and Oyo States in Southwestern Nigeria participated in the study. Heights and weights of 164 girls and 167 boys were measured. Their anthropometric indices, height-for-age (HA), weight-for-height (WH), and weight-for-age (WA) Z-scores determined. The prevalence of stunting (HAZ < -2), wasting (WHZ < -2) and underweight (WAZ < -2) was 38.7%, 13.6%, and 38.7%, respectively when compared to the reference NCHS/WHO standard used for defining stunting, wasting and underweight. Boys were more malnourished than the girls were, but this was not significant (stunting: χ^2 ^= 0.36; df = 1; P = 0.54); (underweight: χ^2 ^= 1.10; df = 1; P = 0.29); and (wasting: χ^2 ^= 0.00; df = 1; P = 0.98) The mean of Z-scores of Height-for-age, Weight-for-age and Weight-for-height in children were -1.502, -1.634 and -0.931 respectively. The SD was 1.52, 1.09 and 1.20 respectively. Using WHO Malnutrition Classification systems, 38.7% of the children were found to be malnourished.

**Conclusion:**

These results indicate high prevalence of malnutrition among settled pastoral Fulani children, possibly due to changes in food habits and lifestyle occasion by the transition from nomadic to sedentary living. We suggest the inclusion of Fulani's settlements in nutritional intervention for these areas.

## Findings

The nomadic Fulani of Northern Nigeria migrating into the Southwest are gradually becoming sedentary [1]. Throughout the region of southwest Nigeria, Fulani settlements are expanding where in some cases resulting in conflicts with host communities [2]. The change from nomadic lifestyle to full sedentary lifestyle is generating changes in their living conditions, food habits, nutrition and health [3,4]. The trend of these changes suggests dire consequences for child nutrition and health as malnutrition arising from both change in food habits and possible inequitable distribution of food within families, although yet undocumented, become rife. Studies on settled pastoral Fulani groups are scanty [5], while the increasing evidence of ill-health, morbidity and mortality in this group calls for the need to provide them with modern health care services [6,7]. Although the pastoral Fulani's are responsible for the provision of animal meats in countries where they are found, their welfare have been largely ignored by government due to inadequate resources since these groups are primarily nomadic. Apart from the studies of Glew *et al*. [8] among semi-nomadic Fulani children in northern Nigeria, there is no information on the nutritional status of settled pastoral Fulani children in Southwest Nigeria necessary to plan appropriate nutritional intervention.

## Methods

The study was carried out in three of the states in southwestern Nigeria. The three states are Ogun, Oyo and Kwara. The three states lie between Latitude 7° 01' and 8° 14' and Longitude 2° 45' and 4° 15'. Oyo and Ogun states are located in humid zone, while Kwara state is in sub-humid zone. The area has a wide range of vegetation zones. The vegetation ranges from fresh water swamp with mangrove forest in the southeast part of Ogun State through diverse forest communities, to the woody Guinea and Sudan savannah in the Northern parts of Oyo and Kwara States. Rainforest now turned disturbed forest, covers a considerable portion of Oyo and Ogun States. A vast portion of the land area in Oyo and Kwara is made up of savannah woodland. The vegetation is dictated mainly by the rainfall pattern. Rainfall ranges from 900 mm in the northern parts of Kwara State, to 1600 mm along the coastal areas of Ogun State. Humidity in the region is between 70 and 95%. The area is inhabited mainly by the Yoruba. The three states were selected because of the presence of a large number of pastoralists that have settled between 1–20 years either in grazing reserves or in locations selected by pastoralists themselves [1].

### Anthropometric measurements

Between March 2003 and December 2004, cross-sectional surveys were conducted in Fulani's settlements as part of a larger study to access the health consequences of lifestyle changes among pastoral populations in southwest Nigeria. During this period anthropometry measurement were taken from Fulani children aged 6 months to 15 years in 61 settlements cutting across the three states. Measurements were performed according to standard procedures of the World Health Organization [9]. Children were weighed wearing light clothes only, and they were measured using a digital scale accurate to 0.5 kg and height was measured to within 0.25 cm using a portable Stadiometer [7]. The ages of children were obtained from their parents or caregivers or sometimes calculated using local events, which could be dated, and linked to important life history episodes. The Z-scores for height-for-age (HAZ), weight-for-height (WHZ) and weight-for-age (WAZ) were calculated using reference data from the US based National Centres for Health Statistics (NCHS) and the World Health Organization [10] in EPI-Info for windows 2000 software (Centres for Disease Control and Prevention, Atlanta, GA). Children were classified as stunted, wasted or underweight if their HAZ, WHZ or WAZ was <-2 respectively. Severely malnourished children were referred to the local health centre near the settlement for care. Other analyses are presented as percentage, mean and standard deviation (SD).

The study was reviewed and approved by the institutional ethical review board of the University of Agriculture, Abeokuta, Nigeria. Written informed consent was obtained from parents or caregivers for each child though interactive meetings and discussions with parents and caregivers.

## Results

A total of 331 settled pastoral children comprising of 167 (50.5%) boys and 164 (49.5%) girls were assessed in Kwara, Oyo and Ogun States. The overall prevalence of stunting was 38.7%, underweight of 38.7% and wasting of 13.6%. Boys were more malnourished than the girls were, but this was not statistically significant (stunting: χ^2 ^= 0.36; df = 1; P = 0.54); (underweight: χ^2 ^= 1.10; df = 1; P = 0.29); and (wasting: χ^2 ^= 0.00; df = 1; P = 0.98) (Table 1).

**Table 1:** Summary of nutritional indicators (Z-scores)

	Height-for-age HAZ (Stunting)	Weight-for-age WAZ (Underweight)	Weight-for-height WHZ (Wasting)
Overall			
No. examined	331	331	310
No. below -2 SD	128	128	45
% below -2 SD	38.7	38.7	14.5
			
Sex			
No.(%) of Girls below -2 SD	58 (35.4%)	56 (34.1%)	19 (11.9%)
No.(%) of Boys below -2 SD	70 (41.9%)	72 (43.1%)	26 (17.2%)

Table 2, 3, &4 shows the nutritional indicators by sex and age group for all indices. Low Height-for-age was highest in 12–23 months age group where 83.3% of children in this age group were stunted (Table 2). Low Weight-for-age was also highest in 12–23 months age group where 75.0% of the children in this age group were underweight (Table 3). Low Weight-for-height was highest in 72–83 age groups where 25.6% of children in this age group were wasting (Table 4). The variations in nutrition indicators within age groups was significantly different for Height-for-Age (χ^2 ^= 35.802; df = 10; P < 0.005) and Weight-for-Age (χ^2 ^= 40.254; df = 10; P < 0.0005) but not significantly different for Weight-for-Height (χ^2 ^= 10.413; df = 10; P = 0.405).

**Table 2:** Prevalence of low height-for-age (stunting) in 331 pastoral Fulani children, by sex and age group.

Age Group (months)	Sex	Number below Cut-off (-2 SD)	Number in age Group	Percentage below Cut-off
6–11.99	Boys	1	1	100.0
	Girls	0	2	0.0
	Combined	1	3	33.3
12–23.99	Boys	11	14	78.6
	Girls	9	10	90.0
	Combined	20	24	83.3
24–35.99	Boys	2	2	100.00
	Girls	6	17	35.3
	Combined	8	19	42.1
36–47.99	Boys	9	16	56.3
	Girls	2	10	20.0
	Combined	11	26	42.3
48–59.99	Boys	3	15	20.0
	Girls	6	16	37.5
	Combined	9	31	29.0
60–71.99	Boys	10	17	58.8
	Girls	2	9	22.2
	Combined	12	26	46.2
72–83.99	Boys	4	22	18.2
	Girls	6	17	35.3
	Combined	10	39	25.6
84–95.99	Boys	5	13	38.5
	Girls	1	25	4.0
	Combined	6	38	15.8
96–107.99	Boys	9	18	50.0
	Girls	1	15	6.7
	Combined	10	33	30.3
108–119.99	Boys	1	7	14.3
	Girls	9	15	60.0
	Combined	10	22	45.5
> 120	Boys	15	42	35.7
	Girls	16	28	57.1
	Combined	31	70	44.3

Total	Boys	70	167	41.9
	Girls	58	164	35.4
	Combined	128	331	38.7

**Table 3:** Prevalence of low weight-for-age (underweight) in 331 pastoral Fulani children, by sex and age group.

Age Group (months)	Sex	Number below Cut-off (-2 SD)	Number in age Group	Percentage below Cut-off
6–11.99	Boys	1	1	100.0
	Girls	0	2	0.0
	Combined	1	3	33.3
12–23.99	Boys	9	14	64.3
	Girls	9	10	90.0
	Combined	18	24	75.0
24–35.99	Boys	2	2	100.00
	Girls	6	17	35.3
	Combined	8	19	42.1
36–47.99	Boys	3	16	18.8
	Girls	3	10	30.3
	Combined	6	26	23.1
48–59.99	Boys	3	15	20.0
	Girls	2	16	12.5
	Combined	5	31	16.1
60–71.99	Boys	5	17	29.4
	Girls	1	9	11.1
	Combined	6	26	23.1
72–83.99	Boys	8	22	36.4
	Girls	8	17	47.1
	Combined	16	39	41.0
84–95.99	Boys	5	13	38.5
	Girls	1	25	4.0
	Combined	6	38	15.8
96–107.99	Boys	12	18	66.7
	Girls	4	15	26.7
	Combined	16	33	48.5
108–119.99	Boys	1	7	14.3
	Girls	10	15	66.7
	Combined	11	22	50.0
> 120	Boys	23	42	54.8
	Girls	12	28	42.9
	Combined	35	70	50.0

Total	Boys	72	167	43.1
	Girls	56	164	34.1
	Combined	128	331	38.7

**Table 4:** Prevalence of low weight-for-height (wasting) in 310 pastoral Fulani children, by sex and age group.

Age Group (months)	Sex	Number below Cut-off (-2 SD)	Number in age Group	Percentage below Cut-off
6–11.99	Boys	0	1	0.0
	Girls	0	2	0.0
	Combined	0	3	0.0
12–23.99	Boys	4	14	28.6
	Girls	1	10	10
	Combined	5	24	20.8
24–35.99	Boys	0	2	0.0
	Girls	4	17	23.5
	Combined	4	19	21.1
36–47.99	Boys	1	16	6.3
	Girls	0	10	0.0
	Combined	1	26	3.8
48–59.99	Boys	3	15	20.0
	Girls	1	16	6.3
	Combined	4	31	12.9
60–71.99	Boys	0	17	0.0
	Girls	2	9	22.2
	Combined	2	26	7.7
72–83.99	Boys	6	22	27.3
	Girls	4	17	23.5
	Combined	10	39	25.6
84–95.99	Boys	5	13	38.5
	Girls	0	25	0.0
	Combined	5	38	13.2
96–107.99	Boys	3	18	16.7
	Girls	3	15	20.0
	Combined	6	33	18.2
108–119.99	Boys	0	7	0.0
	Girls	3	15	20.0
	Combined	3	22	13.6
> 120	Boys	4	26	15.4
	Girls	1	23	4.3
	Combined	5	49	10.2

Total	Boys	26	151	17.2
	Girls	19	159	11.9
	Combined	45	310	14.5

In Table 2, 41.9% of the boys had a low height-for-age or were stunted, while 35.4% of the girls were stunted. There was no significant difference in low height-for-age between boy and girls (P = 0.547). The highest prevalence of stunting of 83.3% was observed among 12–23 months age group, while the lowest of 15.8% was seen in the 84–95 months age group. There was significant difference in stunting among the age groups (P < 0.0005).

In Table 3, 43.1% of the boys had a low weight-for-age or were underweight, compared to 34.1% of the girls. The highest prevalence of underweight of 75.0% was also observed among 12–23 months age group, while the lowest of 16.1% was seen in the 48–59 months age group.

In Table 4, 14.5% of the children were wasting. It also shows that 17.2% of the boys had a low weight-for-height, compared to 11.9% of the girls. The highest prevalence of wasting of 25.6% was recorded in 72–83 months age group, while the lowest of 3.8% was seen in the 36–47 months age group.

The mean of Z-scores of Height-for-age, Weight-for-age and Weight-for-height in the study population were -1.502, -1.634 and -0.931 respectively. The SD was 1.52, 1.09 and 1.20 respectively. Comparing these values to mean and standard deviation of Z-scores of the WHO/NCHS reference population of 0.00 and 1.0, suggests a high prevalence of malnourishment in the population. In a standard population, only 2.3% of the population are expected to fall below -2SD Z-score. Using WHO Malnutrition Classification systems, 38.7% of the children were found to be malnourished.

## Discussion

Nutrition among the pastoral Fulani of Nigeria is traditionally based on milk and dairy products complemented by grains obtained from trade or agro-pastoral production [11]. This study observed a high prevalence of malnutrition of 38.7% among settled Fulani children. This is supported by similar studies in other parts of Nigeria where malnutrition was shown to be prevalence in semi-nomadic Fulani children [7,8,12]. However, the prevalence observed in this study is lower when compared to the Nigerian National average of 40%.

Although the Fulani's eat little, restricting caloric expenditure to about 1700 kcal/day for male adults and 1540 kcal/day for female adults [11]. We observed that, food habits and diets are changing particularly among settled pastoral Fulani. Dairy products, nuts, and fruits are loosing their dominance in the diets of settled Fulani as roots and tubers products are becoming increasingly dominant in their diet compared to their nomadic counterpart. The settled Fulani are increasing their consumption of rice, cassava, yam, wheat, bread soft drinks, canned and processed foods. It will appears that the increasing need for cash income to meet day to day requirements of sedentary living has made it compelling for the Fulani to sell most of their dairy and milk products thereby depriving themselves of adequate protein intake. Sale of dairy and milk products are usually assigned to female members while the male member goes grazing with the herds. This has resulted in less availability of dairy and milk products for their children. These findings support that of Fujita *et al*. [13], where starch was replacing milk in the diet of sedentary pastoralists in northern Kenya. It is then likely that the reduction in the intake of milk products may be one of the factors responsible for the high levels of malnutrition among settled Fulani children in this study. These developments have implications for the nutritional status and health of settled pastoral Fulani children. Contrary to the widely held assumption that settlement of nomadic pastoral Fulani's in grazing reserves, will results in better nutrition, health and living conditions, studies in Kenya among settled pastoralist indeed revealed that settlement diminished nutritional status [14].

In our study, out of the 331 pastoral children assessed, 38.7% were stunted, 38.7% underweight and 13.6% wasting. Although the sample size is small, this is because there is high infant mortality among Fulani pastoral children in Nigeria making it difficult to obtain larger samples [15]. Since we did not assess children of host communities or fully nomadic Fulani children for comparison, it is difficult to determine if the levels of malnutrition observed were due solely to sendentarization or not. Our study did however, point to the fact that the change in food types and habits may have compromised the normally high protein diet often associated with the pastoral Fulani [11]. This suggestion is supported by studies elsewhere, where, malnutrition was three times higher in settled than nomadic children of Rendille in Kenya [16], four times higher in settled than nomadic pastoralist children in Somalia [17] and also more in settled than nomadic pastoralist children in Chad [18].

There was no variation in the level of malnutrition between boy and girl suggesting that both sexes were exposed to same conditions of nutrition and dietary intake. However, there were variation in nutritional indicators between age groups for stunting (HAZ) and underweight (WAZ) indicating that, as settled Fulani children grew older, he or she is likely to become stunted and underweight.

Another possible factor for increasing malnutrition of settled Fulani children may be their living conditions. The Fulani lives in clusters of isolated settlements called "Gaa" with housing made up of mud and grass roofing. Boys who are of age are made to follow the herd from dawn to dusk with little food over long distances and difficult grazing terrains, while girls could hawk dairy products over long distances, to bring in cash for the family. The settlements are located very far from health care services, that can provide health and nutrition education [3]. Other nutritional indicators not assessed in this study but are also important, were iron and Vitamin A deficiencies. Nathan *et al*. [19] reported iron deficiencies among women and children of Kenyan Turkana and Somali semi-nomads, while night blindness, probably due to vitamin A deficiency, was found among Fulani of Mali at the end of the dry season when milk production was lowest [20]. There is therefore the need to undertake a more comprehensive and in-depth analysis of nutrition and health of settled Fulani children populations. Such studies become expedient particularly as there is a growing number of settled Fulani population and sendentarization appear to have become gradually acceptable to the Fulani not as a way of improving the pastoral production system in Nigeria, but because it offers new economic opportunities, such as trading, farming and causal labour. For now, the provision of food supplements and aggressive nutritional education by health workers in settlements are necessary to reduce the currently level of malnutrition observed in this study.

## Conclusion

The findings of this study shows a high prevalence of malnutrition among pastoral Fulani's children settled in southwestern Nigeria. The health authorities in this region should provide nutritional supplement and education in these settlements as more Fulani pastoralist are moving in and settling in southwestern Nigeria.

## Competing interests

The authors declare that they have no competing interests.

## Authors' contributions

UFE and AMO conceptualized the study and designed the study protocols and analysis the collected data. MAD participated in the fieldwork and data collection, UFE and AMO wrote the initial draft manuscript. All authors participated in review of the manuscripts.
